# Geriatric Oncology: From Research to Clinical Practice

**DOI:** 10.3390/cancers12113279

**Published:** 2020-11-05

**Authors:** Nienke A. de Glas

**Affiliations:** Department of Medical Oncology, Leiden University Medical Center, Albinusdreef 2, 2300 RC Leiden, The Netherlands; n.a.de_glas@lumc.nl

The number of older adults with cancer is strongly increasing due to the ageing of Western societies [1]. This poses an enormous burden of older patients on health care systems, who are poorly equipped to deal with this growing number of older patients. The challenge in treating older adults with cancer lies in the large heterogeneity of the population. Ageing-related decline can lead to a decrease in physical functioning, nutritional problems, cognitive deficits or mental health diseases such as depression [1]. In addition, ageing brings an increased risk of concomitant diseases and polypharmacy [2,3]. All these factors are known to be associated not only with a higher mortality risk, but also with an increased risk of treatment toxicity [4,5], thereby complicating treatment decisions in oncology. However, there are very large differences between patients, meaning that calendar age alone is not enough to stratify patients for specific treatments.

Therefore, it is essential to identify these ageing-related factors before starting oncological treatments. The most well-known and validated method to this end is the so-called geriatric assessment, in which all these domains are systematically tested. Many previous studies have shown that a geriatric assessment can adequately identify ageing-related deficits [1,6,7]. This can aid in selecting patients for specific treatments, adapting treatment plans or dosages where necessary, and initiating geriatric interventions to optimize treatments and outcomes [8].

Despite the increasing number of older adults with cancer, the evidence base for the treatment of these patients is limited [9]. This is due to several factors. First, older patients are strongly underrepresented in randomized clinical trials, often due to strict inclusion criteria such as comorbidities, functional status or even calendar age itself [10,11,12]. In addition, it has been shown that even if older patients fulfil the inclusion criteria of clinical trials, they are less frequently included, most likely due to patient or physician preferences [13]. Despite ongoing efforts to increase the trial participation of older adults, there have been almost no improvements in recent years [14]. Alternatively, observational cohort studies and registry data can provide data on outcomes in large groups of (real-world) patients, which can add to the evidence base surrounding older adults with cancer, provided that an adequate research methodology is used.

In this Special Issue of *Cancers*, we invited authors to submit not only clinical studies and implementation studies, but also basic and translational research in older patients with cancer. We were especially interested in studies that focused on further individualizing treatments, such as geriatric assessment studies and biomarker studies. In addition, we invited studies focusing on patient-related outcome measures such as quality of life and functional or cognitive outcomes of treatment, as these outcomes are highly relevant in this population.
